# Engineering of Global Transcriptional Regulators (GTRs) in *Aspergillus* for Natural Product Discovery

**DOI:** 10.3390/jof11060449

**Published:** 2025-06-12

**Authors:** Yujie Zhao, Qing Gong, Huawei Zhang

**Affiliations:** 1 School of Pharmaceutical Sciences, Zhejiang University of Technology, Hangzhou 310014, China; 211122070041@zjut.edu.cn (Y.Z.); 221124070243@zjut.edu.cn (Q.G.); 2 State Key Laboratory of Green Chemical Synthesis and Conversion, Zhejiang University of Technology, Hangzhou 310014, China

**Keywords:** global transcriptional regulators, secondary metabolites, regulatory mechanisms, CRISPR/Cas9, homologous recombination

## Abstract

The *Aspergillus* genus is an important group of filamentous fungi, and the various biological activities of its secondary metabolites (SMs) have great biosynthetic potential. Despite over 4200 SMs having been isolated from *Aspergillus* spp., their metabolic potential remains unexplored due to the presence of numerous silent biosynthetic gene clusters (BGCs) in their genomes. Fortunately, over the last two decades, the global transcriptional regulator (GTR) engineering strategy has emerged as a powerful tool for activating these cryptic BGCs in order to synthesize previously undiscovered SMs from *Aspergillus* spp. This review highlights recent advances in fungal GTR engineering techniques, the regulatory mechanisms of GTRs, and current challenges and future perspectives for their application in natural product discovery in the genus *Aspergillus*.

## 1. Introduction

The genus *Aspergillus*, with 446 known species, stands as one of the most prevalent fungal genera in nature and demonstrates significant biosynthetic potential for producing secondary metabolites (SMs) [1]. According to the Dictionary of Natural Products database, over 4200 SMs produced by *Aspergillus* spp. have been deposited to date [2]. These natural products possess various chemical structures including terpenes, polyketides, peptides, and alkaloids [3,4,5,6]. However, the biosynthetic potential of *Aspergillus* spp. remains underexploited owing to the presence of a large number of silent biosynthetic gene clusters (BGCs) in their genomes. For instance, *A. nidulans* possesses over 50 BGCs, producing significantly fewer characterized SMs under standard cultivation conditions [7,8]. This pronounced disparity between BGC abundance and SM number strongly indicates the prevalence of transcriptionally silent gene clusters awaiting activation. Various strategies have been developed to mine these silent BGCs, such as one strain many compounds (OSMAC) strategies, microbial co-culture, promoter engineering, metabolic shunting, heterologous expression, and transcriptional regulation strategies [9,10,11,12,13].

**Figure 1:**
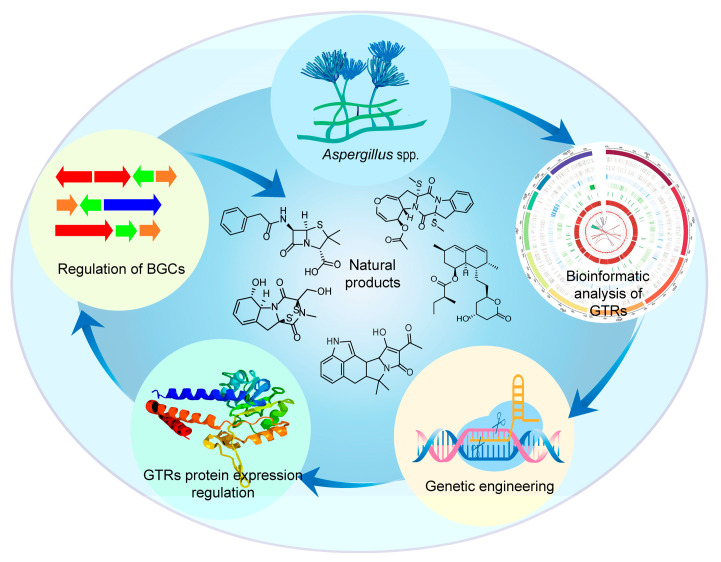
Global transcriptional regulator engineering strategy for secondary metabolite production in *Aspergillus* spp.

Transcriptional regulators, including pathway-specific regulators and global transcriptional regulators (GTRs), can bind to promoters of target genes for gene activation and repression [14]. Gene expression regulation mediated by GTRs is an effective strategy for facilitating natural product discovery (Figure 1). Genes encoding GTRs are generally located outside secondary metabolic gene clusters, and the encoded GTRs directly or indirectly regulate the expression of most genes in *Aspergillus* spp. [15]. Typical GTRs in *Aspergillus* spp. include LaeA, VeA, PacC, CreA, AreA, and CBC (the CCAAT-binding complex). In addition, GTRs such as McrA, LaeB, HbxA/Hbx1, RsmA, RimO (SrrB), Sirtuin E (SirE), StuA and RlcA have been progressively identified and characterized. They regulate various physiological processes in *Aspergillus* spp. such as spore formation, mycelial growth primary metabolism and secondary metabolism, and they can be regulated in response to a variety of environmental signals such as light, pH, carbon source, and nitrogen source [16]. With the continuous innovation and advancement of genetic engineering technology, the clustered regularly interspaced short palindromic repeat/CRISPR-associated protein 9 (CRISPR/Cas9) system, homologous recombination (HR) system-based gene knockout strategies, and gene overexpression strategies using strong promoter or overexpression vectors have been widely used to regulate the expression of GTRs, providing more opportunities to obtain improved strains with secondary metabolic alterations [17,18]. This review systematically summarizes recent advances in various GTR engineering techniques of *Aspergillus* spp. with particular emphasis on elucidating the molecular mechanisms of GTRs and their regulatory effects on SM biosynthesis. It provides a methodological reference for discovering novel natural products and enhancing the production of valuable natural products from *Aspergillus* spp.

## 2. GTR Engineering Approaches

Regulating GTRs expression through methods such as gene knockout and overexpression is an effective strategy for studying their function and regulating downstream gene expression. This section describes key engineering methods for realizing this strategy.

### 2.1. Knockout Strategies for GTR

#### 2.1.1. Knockout of GTR Based on HR

**Figure 2:**
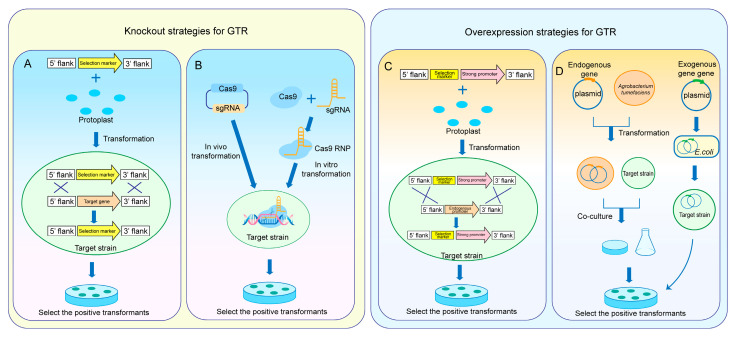
Knockout and overexpression strategies for GTRs. (**A**) Knockout of GTR based on homologous recombination. (**B**) Knockout of GTR based on the CRISPR/Cas9 system. (**C**) Overexpression of GTR using strong promoters. (**D**) Construction of overexpression vectors for GTR expression.

The most common method for the gene knockout of GTRs in *Aspergillus* spp. is using HR to replace the target gene in the strain. This technique uses double-joint polymerase chain reaction (PCR) or fusion PCR to fuse a drug-resistance marker and homologous sequence at both ends into an expression cassette, which is then transformed into the protoplast of the target strain and subsequently screened to construct a successful mutant strain [19,20,21] (Figure 2A).

High efficiency in gene knockout and transformation is key to successfully constructing mutant strains. Non-homologous end joining (NHEJ) and HR are both mechanisms of DNA double-strand break repair [22]. While NHEJ often leads to the random integration of DNA fragments, which can result in transformation failure, disrupting *ku* genes effectively inhibits this error-prone repair mechanism [23,24]. Therefore, constructing *ku*-deficient strains has become a reliable strategy for increasing the success rate of targeted HR. This method was first applied in *A. nidulans* by Nayak et al. to improve the gene targeting efficiency [24]. In *A. niger*, the use of *akuA* (the homologous gene for *ku70*) knockout strains significantly increased the efficiency of HR, resulting in a gene knockout efficiency of over 80% [25]. In *A. fumigatus*, the deletion of *akuB*, the homologous gene for *ku80*, increased the homologous recombination frequency of *pksP* deletion to approximately 80% with 1.5 kb and 2.0 kb flanking regions compared to 5% in the wild-type strain. *akuA* deletion showed similar results [26,27].

The transformation efficiency can also be improved using split-marker technology, which was first developed in yeast [28]. In this method, the selectable marker gene is divided into two overlapping fragments, which replace the target gene and enable gene knockout [29]. In *A. nidulans*, using split-marker-targeting substrates increased the gene targeting efficiency by 2- to 3-fold compared to conventional continuous targeting substrates [30]. In particular, one way to improve the transformation efficiency is by changing the transformation method, such as using *Agrobacterium tumefaciens*-mediated transformation (ATMT), in addition to PEG-mediated transformation and electroporation [31]. ATMT was first applied to filamentous fungi by De Groot et al. A binary vector containing the marker and target genes was constructed and introduced into *Agrobacterium tumefaciens*, which was then co-cultured with the target strains, thereby integrating the target genes into their genome [32]. This transformation system is characterized by a high transformation rate, genetic stability, and a high single-copy integration rate.

#### 2.1.2. Knockout of GTR Based on the CRISPR/Cas9 System

The CRISPR/Cas9 system is an important tool for genome modification. Since its first application as a gene-editing tool in filamentous fungi in 2015, it has been adapted for use in a wide range of filamentous fungal species [33]. CRISPR/Cas9 technology has also been widely used for targeted gene knockout in *Aspergillus* spp. The classic method involves constructing a plasmid containing the Cas9 enzyme and sgRNA to achieve targeted knockout through in vivo transformation [34] (Figure 2B). Recently, the in vitro CRISPR/Cas9 system has also been applied for knocking out GTRs in *Aspergillus* spp. For example, Yuan et al. knocked out *mcrA* in *A. wentii* using an in vitro CRISPR/Cas9 system [35]. In addition, the use of two crRNAs to achieve dual-target cleavage, along with functional protospacer-adjacent motif sequence screening, reduced off-target effects. The targeted knockout efficiency of *mcrA* in *A. wentii* reached 62.5% and was applicable for double-gene knockouts. This method was also successfully applied in *A. melleus* [36].

### 2.2. Overexpression Strategies for GTR

#### 2.2.1. Overexpression of GTR Using Strong Promoters

Strong promoters efficiently recruit RNA polymerase to promote target gene transcription [37]. Therefore, replacing the GTR promoter with a strong promoter can enhance its expression. A classical strategy involves introducing a strong promoter via HR, which is similar to the approach used in HR-based gene knockout (Figure 2C). Promoter types include both natural and synthetic promoters; however, synthetic promoters have not yet been applied to the GTRs expression in *Aspergillus* spp. Commonly used natural promoter types include constitutive promoters and inducible promoters. The glyceraldehyde-3-phosphate dehydrogenase promoter *gpdA* from *A. nidulans* is a representative constitutive promoter, which drives gene stable expression that is relatively unaffected by external conditions [38]. The glucoamylase promoter *glaA*, identified from *A. niger*, is a representative inducible promoter that requires specific inducing factors to function [39]. Recently, the quantitative characterization of filamentous fungal promoters has opened up new possibilities for the discovery of strong promoters [40].

#### 2.2.2. Construction of Overexpression Vectors for GTR Expression

GTRs overexpression can be achieved using overexpression vectors. This strategy involves inserting the target gene into a plasmid and transforming it into the host strain, thereby enhancing target gene expression [41,42] (Figure 2D). Using this technique, Wang et al. constructed the llm1 overexpression vector by cloning the putative methyltransferase gene *llm1* from *A. cristatus* into a plasmid containing the strong promoter *gpdA*. The mutant strain overexpressing *llm1* was subsequently obtained through ATMT, resulting in alterations to its SM profile [43]. This result indicates that this method is applicable for *Aspergillus* spp.

Given the conserved nature of GTRs in *Aspergillus* spp., in addition to overexpressing the endogenous GTR gene in the same strain, compatible heterologous GTRs can also exert global regulatory functions in target strains [44]. Khan et al. used this approach to express the *AnlaeA* gene from *A. nidulans* in *Aspergillus* sp. Z5. [45].

## 3. GTRs in *Aspergillus* spp.

The process of SM biosynthesis is regulated by a complex multilevel molecular network. The GTRs of *Aspergillus* spp. represented by LaeA, VeA, PacC, CreA, AreA, and CBC affect SMs gene expression through different mechanisms, such as the response to environmental signals and chromatin remodeling [15]. Other regulators such as McrA and LaeB have also been successfully identified and confirmed to be GTRs in *Aspergillus* spp. This section summarizes the mechanisms of the different GTRs and the SMs that they regulate.

### 3.1. LaeA

#### 3.1.1. Regulatory Mechanisms of LaeA

LaeA, first identified in *A. nidulans*, functions as a nuclear protein methyltransferase characterized by a conserved S-adenosylmethionine binding site and is conserved across *Aspergillus* spp. [46,47,48]. Although a nuclear localization sequence (NLS) is absent, it is exclusively localized to the nucleus [46].

**Figure 3:**
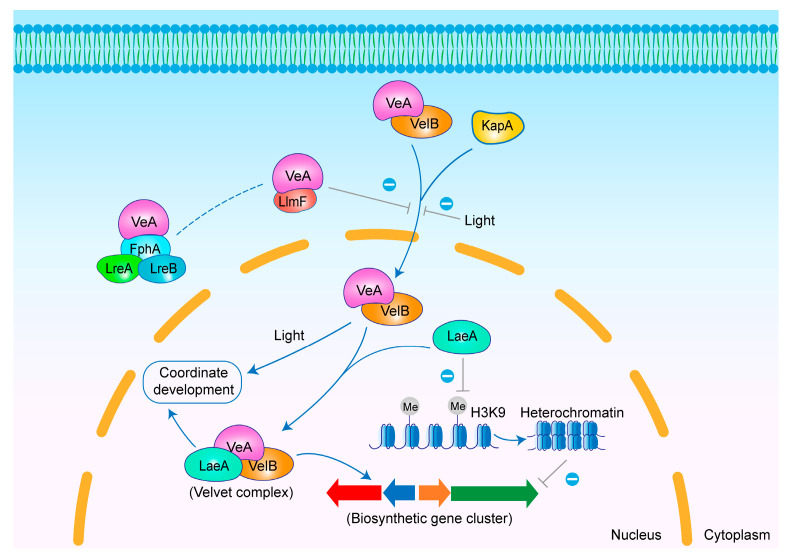
Mechanism of secondary metabolism regulation by LaeA and VeA in *Aspergillus* spp. LlmF, FphA, and light all inhibit the nuclear import of VeA; however, it remains unclear whether LlmF and FphA regulate VeA’s subcellular localization independently or synergistically. In darkness, the VeA-VelB complex is transported into the nucleus and interacts with LaeA to form the velvet complex, which regulates secondary metabolism and development. Additionally, LaeA can regulate secondary metabolism by inhibiting histone methylation.

The regulation of secondary metabolism is the most extensively studied aspect of LaeA. In most cases, LaeA functions as a positive regulator of SMs, and the deletion of *laeA* inhibits the production of many SMs. Chromatin remodeling via histone methylation and velvet complex formation are currently proposed as the primary mechanisms by which LaeA regulates the expression of other genes [49,50,51,52] (Figure 3). It was found that the loss of *laeA* in *A. nidulans* led to a significant increase in histone 3 lysine 9 (H3K9) trimethylation and heterochromatin protein-1 levels, which promoted heterochromatin formation and consequently inhibited sterigmatocystin (ST, **1**) production (Figure S1). Chromatin immunoprecipitation (ChIP) further confirmed that LaeA plays a key role in reversing heterochromatin formation [49]. A study on *A. luchuensis* mut. *Kawachii* with *laeA* knockout demonstrated that LaeA regulates histone 3 lysine 4 (H3K4) and H3K9 methylation to control the expression of the citric acid transporter protein CexA [50]. The loss of laeA in *A. fumigatus* also confirmed this regulatory mechanism, showing the repression of heterochromatin formation at the fungal spore-forming gene *brlA*’s promoter and an increased level of H3K9 trimethylation, suggesting that LaeA regulates *brlA* transcription through chromatin modification [51]. The velvet complex is a protein complex formed in the nucleus by LaeA, VelB, and VeA through which LaeA regulates the expression of other genes [52]. Furthermore, the LaeA regulation of secondary metabolism genes is location-specific, primarily regulating clustered genes in the subtelomeric regions of chromosomes [53,54].

#### 3.1.2. LaeA Regulation-Derived SMs

LaeA plays an essential role in secondary metabolism regulation in *Aspergillus* spp. with examples of it regulating SMs in *Aspergillus* spp. shown in Table 1. Figure S1 provides the structures of all SMs that are regulated.

The deletion of *laeA* significantly suppresses the production of ST (**1**), monacolin J (MONJ, **2**), and the β-lactam antibiotic penicillin G (**3**) in *A. nidulans* FGSC 26; gliotoxin (**4**) in *A. fumigatus* AF293; and the anti-hypercholesterolemic agent lovastatin (**5**) in *A. terreus* ATCC 20542. Conversely, the overexpression of *laeA* significantly enhances penicillin G (**3**) and MONJ (**2**) production in *A. nidulans* FGSC 26 with MONJ (**2**) levels increasing 4-fold. In *A. terreus* ATCC 20542, lovastatin (**5**) production increases 4- to 7-fold, whereas no significant change in ST (**1**) production is observed in *A. fumigatus* AF293 [46]. Similarly, the knockout of *laeA* in *A. fumigatus* AF293 reduces ST (**1**) production to 20% of the wild-type level [55].

The production of aflatoxin B1 and B2 (**6,7**) is suppressed in the laeA deletion mutant of *A. flavus* CA14 by the downregulation of early biosynthetic genes involved in aflatoxin biosynthesis, including *aflR*, *nor1*, and *aflJ* [56]. In the *A. fumigatus* CEA17 *laeA* deletion mutant, key genes (*encA–D*) involved in endocrocin (**8**) biosynthesis are no longer expressed, resulting in the complete loss of endocrocin production [57]. In *A. oryzae* RIB40, the knockout of laeA resulted in a complete loss of kojic acid (**9**) production, while the *laeA* complementation strain restored compound **9** production [58]. The loss of *laeA* in *A. niger* ATCC9029 affected the yields of SMs, resulting in decreased yields of asperrubrol (**10**), atromentin (**11**), and JBIR-86 (**12**) as well as increased yields of BMS-192548 (**13**) and aspernigrin A (**14**) [59]. Biosynthesis of the carcinogenic mycotoxin ochratoxin A (OTA, **15**) is significantly regulated by LaeA, as demonstrated by multiple studies. The deletion of *laeA* in *A. carbonarius* UdL-TA 3.83 led to a decrease in the expression of the key non-ribosomal peptide synthetase (NRPS) involved in OTA (**15**) biosynthesis, leading to a 97% decrease in OTA production relative to the wild-type strain in the dark and a 68.5% decrease in production in the light [60]. The same was found in *A. ochraceus* fc-1 and *A. carbonarius* Ac ITEM 5010 [61,62].

LaeA is a positive regulator of itaconic acid (**16**). In *A. pseudoterreus* ATCC 32359, construction of the *laeA* knockout strain led to a 94% reduction in itaconic acid (**16**) production on glucose/xylose medium compared to that in the wild-type strain. In contrast, *laeA* overexpression increased itaconic acid production by 13% [63]. Simultaneously, LaeA seems to have negative role in the regulation of SM production. For instance, the knockout of *laeA* in *A. flavipes* (507) upregulated the expression of two NRPS-like BGCs and the new piperazine derivatives flavipamide A and B (**17,18**) as well as three known non-ribosomal peptides: N-benzoylphenylalaniny-N-benzoylphenyl-alaninate (**19**), 4′-OMe-asperphenamate (**20**), and cyclic Pro-Gly-Val-Gly-Try (/8-OH, 3-prenyl)-Gly-Trp (**21**) [64].

The overexpression of *laeA* from *A. nidulans* in *Aspergillus* sp. Z5 significantly increased the yields of diorcinol (**22**) and quinolactacin A (**23**). Diorcinol (**22**) exhibited cytotoxic activity against the HCT116 human colon cancer cell line with a half-maximal inhibitory concentration (IC_50_) of 10 µg [45]. LaeA expression enhancement in *A. fumisynnematus* F746 under the control of the strong promoter *alcA* led to the production of a novel metabolite, cyclopiazonic acid (CPA, **24**), which had not previously been identified in *A. fumisynnematus* F746 [65].

Chemical investigation of the *A. versicolor* 0312 *laeA* overexpression mutant led to the identification of a new compound, versicolor A (**25**), as well as four known compounds acetylaranotin (**26**), acety-lapoaranotin (**27**), ergosterol (**28**), and diisobutyl phthalate (**29**); among these, **25** showed cytotoxic activity against the MOLT-4 cell line (IC_50_ = 29.6 µM), while **26** showed good cytotoxic activity against different cell lines such as MOLT-4, CaCo-2, and MCF-7 with IC_50_ values ranging from 7.8 to 19.9 µM [66]. The overexpression of *laeA* in *A. niger* FGSC A1279 resulted in the increased production of three identified compounds including flaviolin (**30**), orlandin (**31**), and kotanin (**32**) [67].

An enhanced expression of *laeA* in *A. terreus* RA2905 activated a silent gene cluster, leading to the production of two small-molecule compounds, dihydroisoflavipucines 1 and 2 (**33**, **34**), which exhibited notable anti-*Vibrio* activity with MIC values ranging from 16 to 64 μg/mL [68]. In the marine fungus *A. niger* L14, the overexpression of *laeA* led to the discovery of five compounds not produced by the wild-type strain, including aspochracin (**35**), JBIR-15 (**36**), sclerotiotide C (**37**), kojic acid (**9**), and penicillic acid (**38**). Among them, JBIR-15 (**36**) exhibited antifungal activity against *Candida albicans* (MIC = 32 μg/mL), while kojic acid (**9**) showed significant DPPH (2,2-diphenyl-1-picrylhydrazyl) radical scavenging activity (IC₅₀ = 5 μg/mL) [69]. The *laeA*-like gene *llm1* was identified in *A. cristatus* CM1303, and the construction of the *llm1* overexpression mutant revealed that llm1 not only positively regulates sexual development but also reduces oxidative stress tolerance to hydrogen peroxide while simultaneously enhancing the production of terpenoids and flavonoids. Unfortunately, the newly generated SMs were not isolated [43].

### 3.2. VeA

#### 3.2.1. Regulatory Mechanisms of VeA

Originally identified in *A. nidulans*, VeA is a member of the velvet family protein that contains a conserved velvet domain and functions as a polyphosphorylated protein whose expression is influenced by its own phosphorylation status [70,71]. Early studies on VeA identified a functional bipartite NLS within its amino acid sequence that is conserved in *Aspergillus* spp. [72].

Compared to LaeA, which is exclusively localized in the nucleus, the subcellular localization of VeA is light-dependent. VeA is predominantly localized in the nucleus under dark conditions, whereas it is mainly found in the cytoplasm in the presence of light [73] (Figure 3). Red and blue light also affect the action of VeA. The red light-sensitive phytochrome FphA modulates VeA activity. VeA interacts with FphA, which then binds to the complex formed by the blue light-sensitive elements LreA and LreB. This cascade ultimately enables VeA to positively regulate sexual development [71,74]. In addition, the LaeA-like methyltransferase LlmF directly interacts with VeA to form a transient complex, thereby impeding the nuclear translocation of VeA and mediating its negative regulation of secondary metabolism in *A. nidulans* [75]. In 2008, the heterotrimeric velvet complex VelB/VeA/LaeA was identified, in which VeA functions as a bridge between VelB and LaeA. In the dark, the NLS is specifically recognized by KapA, which is a homologue of the general nuclear transporter importin α. KapA transports VeA and VelB into the nucleus, where they form a complex with LaeA to jointly regulate sexual development and secondary metabolism in *Aspergillus* spp. Under light conditions, VeA and VelB remain in the cytoplasm and promote asexual development [52].

#### 3.2.2. VeA Regulation-Derived SMs

ST (**1**) and penicillin G (**3**) production in *A. nidulans* FGSC4 is regulated by VeA. The pathway-specific transcription factor AlfR, which controls the mycotoxin ST (**1**), is positively regulated by VeA. The knockout of *veA* leads to reduced transcript levels of *aflR*, thereby further inhibiting ST (**1**) production [76]. Penicillin production is influenced by the delta-(*L*-alpha-aminoadipyl)-*L*-cysteinyl-*D*-valine synthetase gene *acvA*, which positively regulates penicillin biosynthesis. In *A. nidulans* AXB4A2, both the overexpression and deletion of veA inhibit *acvA* expression, thereby reducing penicillin production. These findings suggest that veA negatively regulates penicillin biosynthesis [77]. However, another study demonstrated that VeA positively regulates penicillin production in *A. oryzae* RIB40. This indicates the complexity of the regulatory role of VeA [78].

The knockout of *veA* in *A. flavus* ATCC MYA384 resulted in no further expression of the genes key to aflatoxin biosynthesis, thereby eliminating aflatoxin production. Furthermore, the production of the mycotoxins aflatrem (**39**) and CPA (**24**) was also drastically reduced with CPA (**24**) yields decreasing by 48% in the light and 66% in the dark [79]. To determine the effect of veA on *A. flavus* NRRL 3357, a microarray analysis of *veA* knockout and overexpression mutants revealed that VeA positively regulates genes within the aflatoxin BGC [80]. The deletion of *veA* in *A. fumigatus* CEA10 resulted in a decrease in the expression levels of *gliZ* and *gliP*, genes involved in gliotoxin (**4**) biosynthesis, as well as a 5-fold decrease in gliotoxin (**4**) production compared to the wild-type strain, suggesting that VeA in *A. fumigatus* CEA10 positively regulates gliotoxin (**4**) biosynthesis [81] (Table 2).

The knockout of *veA* in *A. carbonarius* UdL-TA 3.83 led to the downregulation of NRPS, a key enzyme in the biosynthesis of OTA (**15**), resulting in a 90% reduction in OTA (**15**) production compared to the wild-type strain under light conditions. This may be due to VeA’s inability to enter the nucleus and form a complex with LaeA in the presence of light [60]. VeA has been shown to positively regulate conidia production, oxidative stress tolerance, and OTA (**15**) biosynthesis in *A. niger* CICC 41702. The *veA* disruption strain exhibited a significant reduction in OTA production. Quantitative reverse transcription polymerase chain reaction (qRT-PCR) analysis confirmed that VeA influences OTA production by modulating the expression of another key polyketide synthase (PKS) gene within OTA biosynthesis [82].

The analysis of SMs in *A. fumigatus* CEA10 *veA* knockout and overexpression mutants revealed that fumagillin (**40**), fumitremorgin G (**41**), fumigaclavine C (**42**), and glionitrin A (**43**) were among the most significantly altered. In the *veA* knockout mutant, the production of these SMs decreased to approximately 20% of the wild-type level, while in the veA overexpression strain, these SMs were produced at nearly undetectable levels [83]. *A. pachycristatus* NRRL 11440 produces the antifungal drug echinocandin B (ECB, **44**), and the deletion of *Apc.veA* reduced ECB yield by 93% and almost eliminated the by-product ST (**1**). A qRT-PCR analysis revealed a significant reduction in the expression of nine key biosynthetic genes involved in ECB (**44**) and four key biosynthetic genes associated with ST (**1**), demonstrating that VeA positively regulates the production of both ECB and ST [84]. Although VeA generally functions as a positive regulator of SMs, the deletion of *veA* in *A. nidulans* RDIT9.32 activated the production of F9775A (**45**), F9775B (**46**), and orsellinic acid (**47**), suggesting that VeA acts as a negative regulator of these SMs. Further experiments revealed that the gene *gncE*, which encodes GcnE (H3K9 acetyltransferase of the Spt-Ada-Gcn5 acetyltransferase/Ada acetyltransferase complex), was upregulated, indicating that this regulatory effect involves histone H3 acetylation [85].

### 3.3. PacC

#### 3.3.1. Regulatory Mechanisms of PacC

**Figure 4:**
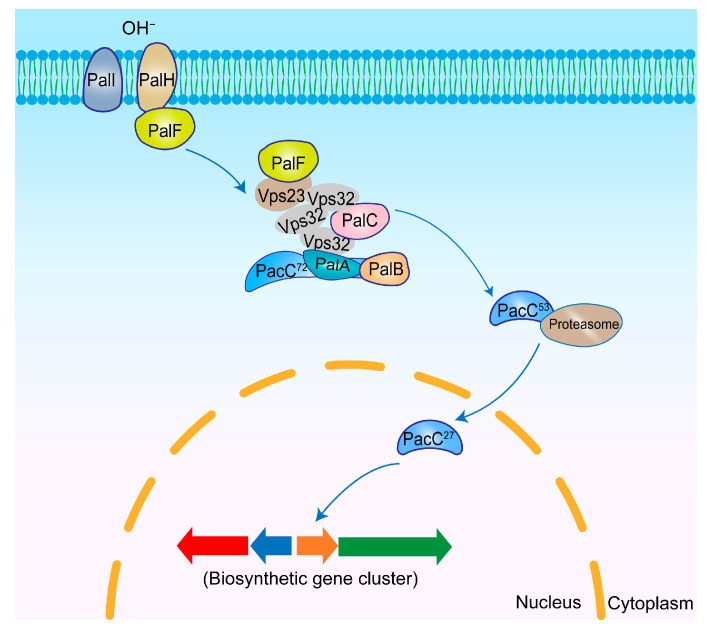
Mechanism of secondary metabolism regulation by PacC in *Aspergillus* spp. PalI and PalH sense the OH⁻ signal and promote PalF ubiquitination. Ubiquitinated PalF then interacts with the ESCRT complex and, with the assistance of PalC, recruits PalA. Subsequently, PalB is incorporated into the complex and cleaves PacC^72^ to produce PacC^53^. PacC^53^ is then further processed by proteases into the activated form PacC^27^, which in turn regulates fungal secondary metabolism.

pH is an essential factor influencing microbial secondary metabolism. The Pal/Rim signaling pathway can regulate gene expression by sensing changes in the extracellular pH. PacC is a key transcriptional regulator of the Pal/Rim signaling pathway with regulatory functions in *Aspergillus* spp. growth, metabolism, and virulence [86]. *pacC* expression is more active at alkaline pH and also activates the transcription of other alkaline-expressed genes [87]. PacC contains three Cys2His2 zinc finger domains. Zinc finger 2 and zinc finger 3 directly bind the consensus sequence 5′-GCCARG-3′, while zinc finger 1 indirectly participates by enhancing the binding ability of zinc finger 3. Additionally, zinc finger 3 contains a nuclear localization signal (NLS) that regulates the subcellular localization of PacC. PacC activates the expression of target genes by directly binding to the consensus sequence [88,89]. Ambient pH alters the active form and localization of PacC, which, in turn, affects its ability to transcriptionally regulate other genes [90]. PacC’s C-terminal region has been shown to negatively regulate its activity, rendering the full-length PacC protein (72 kDa) inactive [91] (Figure 4).

PalI, PalH and PalF are three integral components of the pH-sensing signaling complex localized to the *A. nidulans* plasma membrane [92]. Under alkaline conditions, PalI and PalH at the membrane sense the extracellular OH⁻ signal and subsequently promote PalF ubiquitination. Ubiquitinated PalF then facilitates the recruitment of the ESCRT-I (endosomal sorting complex required for transport I) component Vps23, which, in turn, recruits additional ESCRT required for transport [93]. The ESCRT-III core subunit Vps32 interacts with PalC (72 Ka), leading to the subsequent recruitment of PalA. The complex formed by PalA and full-length PacC associates with the calpain-like protease PalB, resulting in the proteolytic cleavage of the C-terminal domain of PacC and generation of PacC^53^ (53 kDa), which is further processed by proteasome-mediated partial hydrolysis into the active form PacC^27^ (27 kDa), which contains a DNA-binding domain and functions as a transcriptional regulator, activating alkaline-expressed genes while repressing acid-expressed genes [94,95,96]. The active form of PacC also affects its subcellular localization. When in its full-length form, it is mainly localized in the cytoplasm, whereas the C-terminally truncated active form, PacC^27^, is predominantly localized in the nucleus [97].

#### 3.3.2. PacC Regulation-Derived SMs

PacC is involved in secondary metabolic processes in *Aspergillus* spp. In *A. nidulans*, PacC can directly bind to the sequence between *acvA* and *ipnA*, thereby modulating *ipnA* expression and ultimately governing penicillin biosynthesis [98]. A series of PacC gain-of-function strains were obtained in *A. nidulans* by mimicking alkaline mutations, and both their penicillin yield and *ipnA* gene expression levels were significantly higher than those of the wild-type strain [99]. O-methylsterigmatocystin (**48**) in *A. parasiticus* RHN1 is regulated by PacC, and it is reduced in strains with deletion of the PacC binding site in the *aflR* promoter [100]. When *AopacC* was knocked out of *A. ochraceus* fc-1, both acidic and alkaline conditions significantly reduced OTA (**15**) production and the expression of the related biosynthetic gene *Aopks* compared to that in neutral conditions [101]. PacC also modulates OTA (**15**) biosynthesis in *A. carbonarius* NRRL 368, as proven by the *pacC* deletion strain completely ceasing OTA production at pH 7.0 [102] (Table 3).

### 3.4. CreA

#### 3.4.1. Regulatory Mechanisms of CreA

**Figure 5:**
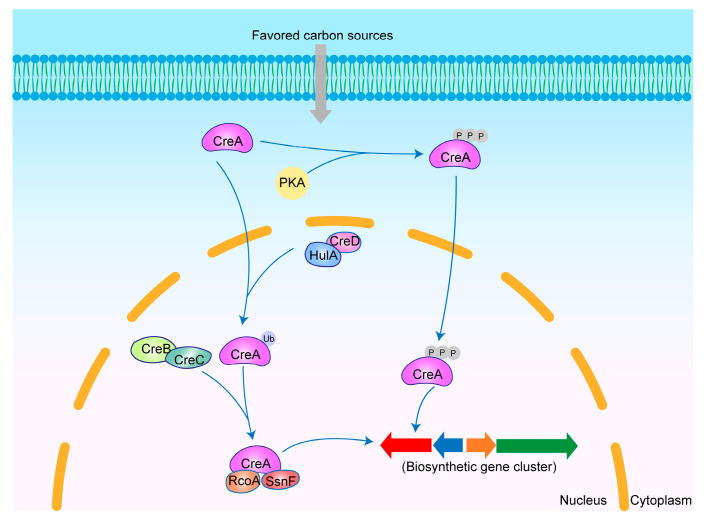
Mechanism of secondary metabolism regulation by CreA in *Aspergillus* spp. CreA is ubiquitinated by the CreD–HulA complex and subsequently deubiquitinated by the CreB–CreC complex. The deubiquitinated CreA then associates with RcoA and SsnF to regulate secondary metabolism. In addition, favored carbon sources can further govern secondary metabolism by promoting CreA phosphorylation.

CreA is a major regulator of carbon source utilization in *Aspergillus* spp. and has been shown to be a repressor protein containing two C2H2-like zinc finger domains, which can regulate the expression of other genes by binding to the consensus sequence 5′-SYGGRG-3′ [109,110]. CreA regulates gene expression both directly and indirectly. In *A. nidulans*, CreA can indirectly regulate the expression of other xylanase genes by directly binding to the consensus sequence in *xlnR*’s promoter region [111]. In addition, changes in carbon source availability indirectly influence the CreA-mediated regulation of other genes. Carbon catabolite repression (CCR) is a mechanism by which microorganisms are able to preferentially utilize favored carbon sources (such as D-glucose, D-fructose, or D-xylose) during microbial growth, thereby inhibiting the utilization of other carbon sources. CreA was shown to be a transcriptional repressor involved in this mechanism. Alteration of the carbon source modulates the transcript level of *creA* [112]. In the presence of favored carbon sources, the *creA* expression levels increase, leading to the negative regulation of other genes. Conversely, under repressing carbon source conditions, creA expression is suppressed, resulting in de-repression [113] (Figure 5).

The nuclear localization of CreA is independent of its activity but is affected by different carbon sources. CreA localizes to the nucleus in the presence of favored carbon sources and to the cytoplasm when favored carbon sources are absent [114]. CreA activation is influenced by both ubiquitination and phosphorylation. In the presence of favored carbon sources, the complex formed by the interaction between the ubiquitin ligase HulA and the arrestin-like protein CreD mediates CreA ubiquitination [115]. Subsequently, the CreB–CreC deubiquitination complex, composed of the deubiquitinating enzyme CreB and the scaffold protein CreC, mediates CreA deubiquitination, and the deubiquitinated CreA then associates with the corepressors SsnF and RcoA, resulting in the activation of CreA’s repressive function [116,117,118]. In addition, favored carbon sources induce CreA phosphorylation, mediated by protein kinase A (PKA), which also contributes to CreA activation [119,120]. Furthermore, autogenous regulation is also an important mode of *creA* expression, and CreA negatively regulates its own expression [121].

#### 3.4.2. CreA Regulation-Derived SMs

Aflatoxin B1 (**6**) biosynthesis in *A. flavus* CA14PTS is regulated by CreA. In the *creA* overexpressing mutant, aflatoxin B1 (**6**) production was reported to increase from 0.096 μg/g to 0.105 μg/g when grown in complete medium. In contrast, the deletion of *creA* resulted in the almost complete loss of aflatoxin B1 (**6**) production [103]. In *A. ochraceus* fc-1, the knockout of *creA* inhibits conidiation and OTA (**15**) production with different carbon sources [104]. Analysis of the CreA regulatory network revealed that more than 35% of the genes encoding transcription factors in the *A. nidulans* genome are under CreA control. This highlights the global regulatory role of CreA [122] (Table 3).

### 3.5. AreA

#### 3.5.1. Regulatory Mechanisms of AreA

**Figure 6:**
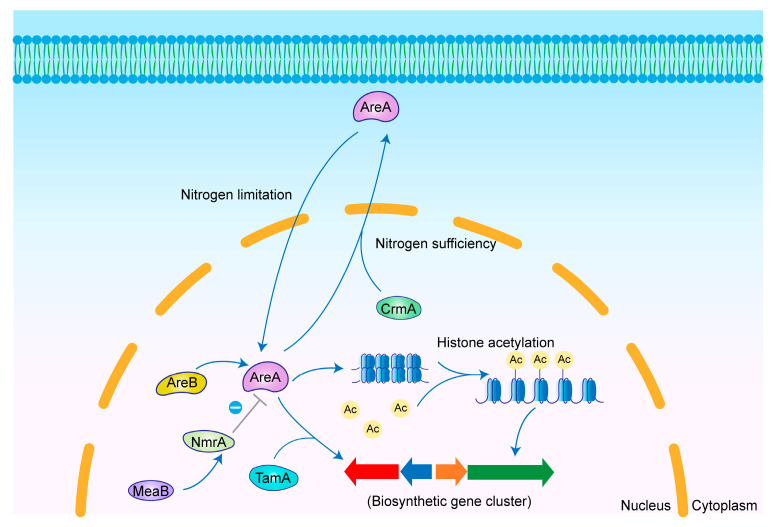
Mechanism of secondary metabolism regulation by AreA in *Aspergillus* spp. Under nitrogen limitation, AreA undergoes nuclear import and subsequently activates silent BGCs by directly binding to secondary metabolite genes or by facilitating chromatin remodeling through promoted histone acetylation.

AreA is a GATA-type positive transcriptional regulator that contains a C-terminal zinc finger domain and a DNA-binding domain recognizing the consensus sequence. Early studies identified AreA as the major regulatory protein in nitrogen metabolite repression (NMR). It not only indirectly regulates the expression of other genes by influencing nitrogen source utilization but also directly regulates gene expression by binding to the consensus sequence [123,124,125]. Furthermore, AreA had been shown to regulate other genes through histone H3 acetylation-mediated chromatin remodeling [126,127] (Figure 6). Additional regulatory factors were shown to interact with AreA to achieve indirect regulation. For example, the co-activator TamA interacts with AreA to promote gene expression [128]. Another GATA factor in the NMR pathway, AreB, has been shown to positively regulate AreA expression [129]. Furthermore, the NMR pathway repressor NmrA binds to AreA to inhibit its activity, and bZIP transcription factor MeaB indirectly regulates AreA activity by activating NmrA expression [130,131].

The nuclear localization of AreA is affected by the nitrogen source. In *A. nidulans*, there are five classical NLSs in the AreA sequence as well as an atypical bipartite NLS within the DNA-binding domain. These six NLSs act together to regulate the nuclear localization of AreA [132]. In the NMR mechanism, nitrogen starvation causes AreA to be primarily localized in the nucleus, where it activates genes dependent on AreA. Upon detection of the nitrogen signal, CrmA facilitates the nuclear export of AreA, leading to its rapid accumulation in the cytoplasm. This suggests that AreA acts as a positive regulator in NMR [133].

#### 3.5.2. AreA Regulation-Derived SMs

Most studies on AreA have focused on its role in NMR, while other studies have suggested that AreA also plays a regulatory role in *Aspergillus* spp. secondary metabolism. The deletion of *areA* in *A. terreus* SBUG844 results in a reduced expression of *terR*, a specific activator of terrein (**49**), which in turn decreases terrein (**49**) biosynthesis. The loss of *areA* in *A. terreus* SBUG844 also inhibits growth on media where methionine is the only nitrogen source [105]. The nitrogen source affects aflatoxin B1 (**6**) production in *A. flavus* SRRC1709. AreA, as a major regulator of nitrogen metabolism, can further affect aflatoxin B1 metabolism by influencing nitrogen metabolism, and it was found that the presence of glutamine, the optimal nitrogen source for aflatoxin B1 synthesis, with either the overexpression or deletion of *area*, resulted in increased aflatoxin B1 production [106] (Table 3).

### 3.6. CBC

#### 3.6.1. Regulatory Mechanisms of CBC

**Figure 7:**
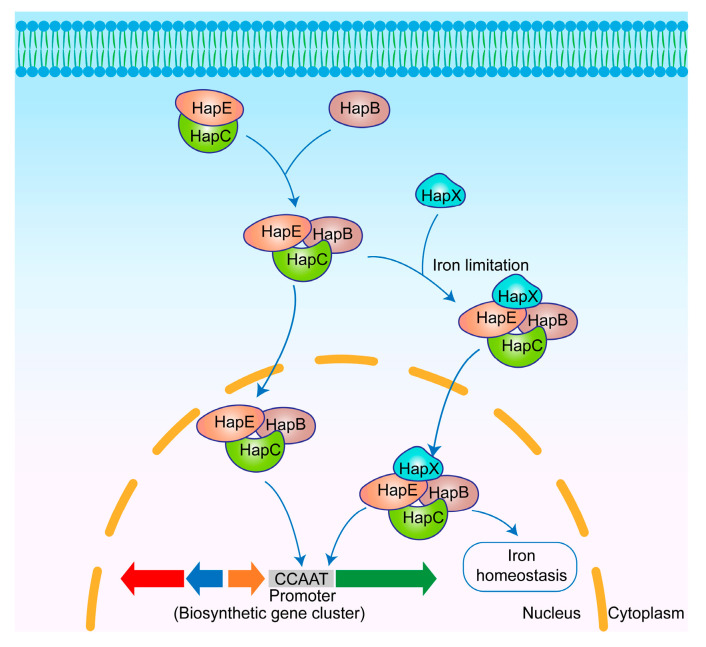
Mechanism of secondary metabolism regulation by CBC in *Aspergillus* spp. The HapE–HapC complex associates with HapB to form CBC, which translocates into the nucleus and regulates the expression of biosynthetic genes by binding to the CCAAT box within promoters. Furthermore, it can form a complex with HapX to regulate secondary metabolism and iron homeostasis.

CBC is a transcriptional regulator comprising three core subunits: HapB, HapC, and HapE. CBC binds to the CCAAT box in the promoter regions of target genes, thereby regulating their transcription [134]. The bZIP-type transcriptional regulator HapX is activated under iron-restricted conditions and subsequently interacts with CBC to form a complex that co-regulates genes involved in iron homeostasis in *Aspergillus* spp. [135]. HapC and HapE, which do not possess NLS, depend on their interaction with the NLS-bearing HapB to assemble the CBC. CBC is subsequently imported into the nucleus and performs its regulatory roles [136,137] (Figure 7). It has been demonstrated that the knockout of *hapB* resulted in a rate of 46.3% for differentially expressed genes in *A. fumigatus*, further indicating the global regulatory role of CBC [138].

#### 3.6.2. CBC Regulation-Derived SMs

In *A. nidulans* MH8193, penicillin biosynthesis is regulated by CBC. The deletion of *hapC* significantly reduces *ipnA* and *aat* (the gene encoding isopenicillin N acyltransferase) expression and also decreases penicillin production by approximately 30% [107]. In *A. fumigatus* A1160P, *hapC* deletion mutants showed a 2.4-fold increase in ergosterol levels, and *hapE* loss-of-function mutations increased ergosta-5,7,24(28)-trien-3β-ol (**50**) and episterol (**51**) production [108] (Table 3). These results revealed that CBC negatively regulates sterol biosynthesis and positively regulates penicillin biosynthesis.

### 3.7. Other GTRs

McrA, a GTR containing the Zn(II)₂Cys₆ structural domain, is a negative regulator of fungal SMs identified through a genetic screening approach in *A. nidulans*. In the *A. nidulans* FGSC A4 *mcrA* knockout mutant, the production of nine SMs was found to be upregulated, and two new compounds (**52**,**53**) were identified [139]. However, its regulatory mechanisms have not been precisely studied. The knockout of *mcrA* in *A. nidulans* FGSCA442 upregulated the production of 15 SMs and led to the biosynthesis of a new cichorine intermediate (**54**) [140]. The deletion of *mcrA* in *A. wentii* IMI 49129 upregulates the production of 15 SMs and led to the identification of nine novel chemicals (**55**–**63**), while knockout of the polyketide synthase gene resulted in the production of aspergillus acids B (**64**) and E (**65**) [35]. In the *mcrA* deletion mutant of *A. melleus* IMV 01140, the neoaspergillic acid (**66**) and neohydroxyaspergillic acid (**67**) yields were increased by 1.7- and 1.6-fold, respectively [36]. In the *A. oryzae* NSAR1 *mcrA* knockout mutant, although no obvious production of new SM was observed, the production of kojic acid (**9**) increased from 1.23 g/L to 2.52 g/L [141]. The overexpression of *mcrA* in *A. nidulans* FGSC4 completely inhibited ST (**1**) biosynthesis, highlighting the role of McrA as a negative regulator [142] (Table 4).

A new GTR, LaeB, was identified through a forward genetic screening approach in *A. nidulans*. LaeB has a significant effect on SMs, and the production of ST (**1**) in *A. nidulans* BTP69 and aflatoxin in *A. flavus* NRRL3357 was abolished as a result of *laeB* knockout [143]. The deletion of *laeB* activated silent BGCs in *A. nidulans* RJMP1.49 and led to the production of four new substances (**68**–**71**) and four known metabolites (**72**–**75**). This demonstrates the potential of LaeB to unlock the production of cryptic natural products in *Aspergillus* spp. [144].

HbxA/Hbx1 is a class of GTRs with homeobox protein domains. In *A. flavus* CA14, CPA (**24**), aflatrem (**39**), and aflatoxin cannot be detected after the deletion of *hbx1* [145]. In contrast, the *A. nidulans* FGSC4 *hbxA* knockout mutant increased ST (**1**) production, suggesting that HbxA acts as a negative regulator of ST biosynthesis [146]. The mutagenic screening of *laeA* deletion *A. nidulans* RJMP1.49 strain led to the identification of the bZIP-type GTR RsmA [154]. RsmA positively regulates ST (**1**) production in *A. nidulans* RJMP1.49 [147]. The overexpression of *rsmA* in *A. fumigatus* AF293 increased gliotoxin (**4**) and cyclo(L-Phe-L-Ser) (**76**) production [148]. AflrsmA, a homolog of RsmA from *A. flavus* CA14, was shown to regulate the production of the secondary metabolite aflatoxin B1 (**6**) through the oxidative stress pathway [149]. The APSES family transcription factor StuA was originally recognized as a key protein involved in filamentous fungal development [155,156]. Recent studies on StuA in *A. terreus* RA2905 have demonstrated that the deletion of *stuA* significantly increases the yield of the anticancer drug terrein (**49**) by 21-fold compared to that from the wild-type strain while reducing the content of the by-product butyrolactones [150].

The NAD+-dependent histone deacetylase SirE has been identified as a GTR associated with epigenetic regulation. SirE positively regulates ST (**1**) biosynthesis in *A. nidulans* A26 and negatively regulates aflatoxin production in *A. flavus* CA14 PTS [151,152]. The novel chromatin-associated GTR, RimO, was discovered through an RNA sequencing (RNA-seq) analysis of *A. nidulans* FGSC4. RimO has been shown to positively regulate the starvation-induced production of SMs. RimO deletion led to ST (**1**) being nearly undetectable, while the RimO overexpression mutant not only promoted the biosynthesis of ST but also enhanced the production of penicillin G (**3**) [153]. The recently identified RlcA is a regulator of light sensing and chromatin remodeling, and a transcriptome analysis revealed that RlcA regulates more than one third of the genes in *A. nidulans* [157]. Although there is currently no specific research on the secondary metabolism regulation by RlcA, it is speculated that RlcA may indirectly influence the expression of secondary metabolism genes through chromatin remodeling [158].

## 4. Conclusions and Perspectives

The regulatory network of GTRs is complex, and regulating GTR expression can affect SMs production in *Aspergillus* spp. In this review, we summarize engineering approaches for the knockout and overexpression of GTRs in *Aspergillus* spp. LaeA, VeA, PacC, CreA, AreA, and CBC are typical GTRs in *Aspergillus* spp. We have elucidated in detail the possible mechanisms by which they exert their regulatory functions based on previous studies and have described the SMs regulated by them. In addition, further exploration of the regulatory network of GTRs has led to the identification of several novel GTRs. These new GTRs were identified either through genetic screening or from previously known transcriptional regulators that were revealed to function as potential GTRs. These GTRs have been used to discover novel compounds, including flavipamide A and B (**17**,**18**), versicolor A (**25**), 3-methoxyporriolide (**70**), and 7-methoxyporriolide (**71**). Additionally, the production of some natural product drugs, such as penicillin G (**3**) and lovastatin (**5**), has also been increased.

Multi-omics technologies have been applied for GTR identifications and investigations into their regulatory mechanisms, and a deeper understanding of the GTRs regulatory networks will promote the natural products discovery [157,159]. Furthermore, with the continuous development of genetic engineering technologies, it is important to improve gene editing precision and to apply emerging technologies more broadly to the regulation of GTR expression in *Aspergillus* spp. Efficient genetic targeting methods can accelerate gene overexpression or knockout and enable the rapid verification of gene functions. Currently, some recently identified GTRs, such as RlcA, have not been applied for the activation of silent BGCs, despite their predicted potential to regulate secondary metabolism. In the future, more attention could be directed toward exploring the regulatory potential of these factors in secondary metabolism.

## Figures and Tables

**Table 1:** Examples of secondary metabolites regulated by LaeA in *Aspergillus* spp.

*Aspergillus* Strain	Deletion or Overexpression	Upregulated SMs	Downregulated SMs	Ref.
*A. nidulans* FGSC 26	Deletion	-	Sterigmatocystin (ST, **1**), monacolin J (MONJ, **2**), and penicillin G (**3**)	[46]
	Overexpression	Penicillin G (**3**) and MONJ (**2**)	-	
*A. fumigatus* AF293	Deletion	-	Gliotoxin (**4**)	
*A. terreus* ATCC 20542	Deletion	-	Lovastatin (**5**)	
	Overexpression	Lovastatin (**5**)	-	
*A. fumigatus* AF293	Deletion	-	ST (**1**)	[55]
*A. flavus* CA14	Deletion	-	Aflatoxin B1 (**6**) and aflatoxin B2 (**7**)	[56]
*A. fumigatus* CEA17	Deletion	-	Endocrocin (**8**)	[57]
*A. oryzae* RIB40	Deletion	-	Kojic acid (**9**)	[58]
*A. niger* ATCC9029	Deletion	BMS-192548 (**13**) and aspernigrin A (**14**)	Asperrubrol (**10**), atromentin (**11**) and JBIR-86 (**12**)	[59]
*A. carbonarius* UdL-TA 3.83	Deletion	-	Ochratoxin A (OTA, **15**)	[60]
*A. ochraceus* fc-1	Deletion	-	OTA (**15**)	[61]
*A. carbonarius* Ac ITEM 5010	Deletion	-	OTA (**15**)	[62]
*A. pseudoterreus* ATCC 32359	Deletion	-	Itaconic acid (**16**)	[63]
*A. flavipes* (507)	Deletion	Flavipamide A and B (**17,18**), *N*-benzoylphenylalaniny-*N*-benzoylphenyl-alaninate (**19**), 4′-OMe-asperphenamate (**20**), cyclic Pro-Gly-Val-Gly-Try (/8-OH, 3-prenyl)-Gly-Trp (**21**)	-	[64]
*Aspergillus* sp. Z5	Overexpression	Diorcinol (**22**) and quinolactacin A (**23**)	-	[45]
*A. fumisynnematus* F746	Overexpression	Cyclopiazonic acid (**24**)	-	[65]
*A. versicolor* 0312	Overexpression	Versicolor A (**25**), acetylaranotin (**26**), acetylapoaranotin (**27**), ergosterol (**28**) and diisobutyl phthalate (**29**)	-	[66]
*A. niger* FGSC A1279	Overexpression	Flaviolin (**30**), orlandin (**31**), and kotanin (**32**)	-	[67]
*A. terreus* RA2905	Overexpression	Dihydroisoflavipucines 1 and 2 (**33**, **34**)	-	[68]
*A. niger* L14	Overexpression	Aspochracin (**35**), JBIR-15 (**36**), sclerotiotide C (**37**), kojic acid (**9**), and penicillic acid (**38**)	-	[69]

**Table 2:** Examples of secondary metabolites regulated by VeA in *Aspergillus* spp.

*Aspergillus* Strain	Deletion or Overexpression	Upregulated SMs	Downregulated SMs	Ref.
*A. nidulans* FGSC4	Deletion	-	ST (**1**) and penicillin G (**3**)	[76]
*A. nidulans* AXB4A2	Deletion	-	Penicillin G (**3**)	[77]
	Overexpression	-	Penicillin G (**3**)	
*A. oryzae* RIB40	Deletion	-	Penicillin G (**3**)	[78]
*A*. *flavus* ATCC MYA384	Deletion	-	Aflatoxin, aflatrem (**39**) and CPA (**24**)	[79]
*A. fumigatus* CEA10	Deletion	-	Gliotoxin (**4**)	[81]
*A. carbonarius* UdL-TA 3.83	Deletion	-	OTA (**15**)	[60]
*A. niger* CICC 41702	Deletion	-	OTA (**15**)	[82]
*A. fumigatus* CEA10	Deletion	-	Fumagillin (**40**), fumitremorgin G (**41**), fumigaclavine C (**42**), and glionitrin A (**43**)	[83]
	Overexpression	-	fumagillin (**40**), fumitremorgin G (**41**), fumigaclavine C (**42**), and glionitrin A (**43**)	
*A. pachycristatus* NRRL 11440	Deletion	-	Echinocandin B (ECB,**44**) and ST (**1**)	[84]
*A. nidulans* RDIT9.32	Deletion	F9775A (**45**), F9775B (**46**), and orsellinic acid (**47**)	-	[85]

**Table 3:** Examples of secondary metabolites regulated by PacC, CreA, AreA, and CBC in *Aspergillus* spp.

GTRs	*Aspergillus* Strain	Deletion or Overexpression	Upregulated SMs	Downregulated SMs	Ref.
PacC	*A. nidulans*	Overexpression	Penicillin G (**3**)	-	[99]
	*A. parasiticus* RHN1	Deletion of the PacC binding site	-	O-methylsterigmatocystin (**48**)	[100]
	*A. ochraceus* fc-1	Deletion	-	OTA (**15**)	[101]
	*A. carbonarius* NRRL 368	Deletion	-	OTA (**15**)	[102]
CreA	*A. flavus* CA14PTS	Overexpression	Aflatoxin B1 (**6**)	-	[103]
		Deletion	-	Aflatoxin B1 (**6**)	
	*A. ochraceus* fc-1	Deletion	-	OTA (**15**)	[104]
AreA	*A. terreus* SBUG844	Deletion	-	Terrein (**49**)	[105]
	*A. flavus* SRRC1709	Overexpression	Aflatoxin B1 (**6**)	-	[106]
		Deletion	Aflatoxin B1 (**6**)		
CBC	*A. nidulans* MH8193	Deletion of *hapC*	-	Penicillin (**3**)	[107]
	*A. fumigatus* A1160P^+^	Deletion of *hapE*	Ergosta-5,7,24(28)-trien-3β-ol (**50**) and episterol (**51**)	-	[108]

**Table 4:** Examples of secondary metabolites regulated by other GTRs in *Aspergillus* spp.

GTRs	*Aspergillus* Strain	Deletion or Overexpression	Upregulated SMs	Downregulated SMs	Ref.
McrA	*A. Nidulans* FGSC A4	Deletion	1,3-Dihydro-6-hydroxy-4-methoxy-5-methyl-1-oxo-2H-isoindole-2-pentanoic acid (**52**), 4-[hydroxy(4-hydroxy-2-methoxy-3,6-dimethylphenyl)methoxy]-2-methoxy-3,6-dimethylbenzaldehyde (**53**)	-	[139]
	*A. nidulans* FGSCA442	Deletion	Cichorine intermediate (**54**)	-	[140]
	*A. wentii* IMI 49129	Deletion	Emodin (**55**), physcion (**56**), sulochrin (**57**), 14-O-demethylsulochrin (**58**), physcion bianthrone (**59**), (trans)-emodin bianthrone (**60**), (cis)-emodin bianthrone (**61**), (trans)-emodin physcion bianthrone (**62**), (cis)-emodin physcion bianthrone (**63**), aspergillus acid B (**64**), and aspergillus acid E (**65**)	-	[35]
	*A. melleus* IMV 01140	Deletion	Neoaspergillic acid (**66**), and neohydroxyaspergillic acid (**67**)	-	[36]
McrA	*A. oryzae* NSAR1	Deletion	Kojic acid (**9**)	-	[141]
	*A. nidulans* FGSC4	Overexpression	-	ST (**1**)	[142]
LaeB	*A. Nidulans* BTP69	Deletion	-	ST (**1**)	[143]
	*A. flvus* NRRL3357	Deletion	-	Aflatoxin	
LaeB	*A. nidulans* RJMP1.49	Deletion	Gibellulin C (**68**), gibellulin D (**69**), 3-methoxyporriolide (**70**), 7-methoxyporriolide (**71**), porriolide (**72**), cichorine (**73**), asperthecin (**74**), microperfuranone (**75**)	ST (**1**)	[144]
HbxA/Hbx1	*A*. *flavus* CA14	Deletion of *hbx1*	-	CPA (**24**), aflatrem (**39**) and aflatoxin	[145]
	*A. nidulans* FGSC4	Deletion of *hbxA*	ST (**1**)	-	[146]
RsmA	*A. nidulans* RJMP1.49	Overexpression	ST (**1**)	-	[147]
	*A. fumigatus* AF293	Overexpression	Gliotoxin (**4**) and cyclo(L-Phe-L-Ser) (**76**)	-	[148]
	*A. flavus* CA14	Overexpression	Aflatoxin B1 (**6**)	-	[149]
StuA	*A. terreus* RA2905	Deletion	Terrein(**49**)	Butyrolactones	[150]
SirE	*A. nidulans* A26	Deletion	-	ST (**1**)	[151]
	*A. flavus* CA14 PTS	Deletion	Aflatoxin	-	[152]
RimO	*A. nidulans* FGSC4	Overexpression	ST (**1**) and penicillin G (**3**)	-	[153]
		Deletion	-	ST (**1**)	

## Data Availability

The data presented in this study are available in the Supplementary Materials.

## Supplementary Materials

The following supporting information can be downloaded at https://www.mdpi.com/article/10.3390/jof11060449/s1, Figure S1: Structures of secondary metabolite 1-76 regulated by global transcriptional regulators in *Aspergillus* spp.

## Abbreviations

The following abbreviations are used in this manuscript:

ATMT	*Agrobacterium tumefaciens*-mediated transformation
BGCs	biosynthetic gene clusters
Cas9	CRISPR-Associated Protein 9
CBC	the CCAAT-binding complex
CCR	carbon catabolite repression
ChIP	chromatin immunoprecipitation
CPA	cyclopiazonic acid
CRISPR	clustered Regularly Interspaced Short Palindromic Repeats
DPPH	2,2-diphenyl-1-picrylhydrazyl
ECB	echinocandin B
ESCRT	endosomal sorting complex required for transport
GTRs	global transcriptional regulators
HR	homologous recombination
H3K4	histone 3 lysine 4
H3K9	histone 3 lysine 9
IC_50_	half maximal inhibitory concentration
MIC	minimum Inhibitory Concentration
MONJ	monacolin J
NHEJ	non-homologous end joining
NLS	nuclear localization sequence
NMR	nitrogen metabolite repression
NRPS	non-ribosomal peptide synthetase
OSMAC	one strain many compounds
OTA	ochratoxin A
PCR	polymerase chain reaction
PKA	protein kinase A
PKS	polyketide synthase
qRT-PCR	quantitative reverse transcription polymerase chain reaction
RNA-seq	RNA sequencing
SirE	sirtuin E
SMs	secondary metabolites
ST	sterigmatocystin
