# Supplementary material for: Engineering of Global Transcriptional Regulators (GTRs) in Aspergillus for Natural Product Discovery
Source: J Fungi (Basel). 2025 Jun 12;11(6):449. doi: 10.3390/jof11060449 (PMC12193740; doi:10.3390/jof11060449)
Supplement: Supplementary file 1 [file jof-11-00449-s001.zip › Supplementary Materials/Supplementary Materials.pdf]

Supplementary Materials:

Engineering of global transcriptional regulators (GTRs) in *Aspergillus* for natural product discovery

Yujie Zhao [1,2], Qing Gong [1,2] and Huawei Zhang [1,2,*]

[1] School of Pharmaceutical Sciences, Zhejiang University of Technology, Hangzhou 310014, China; 211122070041@zjut.edu.cn (Y.Z.); 221124070243@zjut.edu.cn (Q.G.)
[2] State Key Laboratory of Green Chemical Synthesis and Conversion, Zhejiang University of Technology, Hangzhou 310014, China
* Correspondence: hwzhang@zjut.edu.cn; Tel.: +86-571-8832-0913

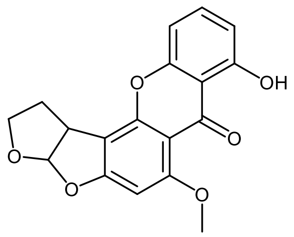

1. Sterigmatocystin

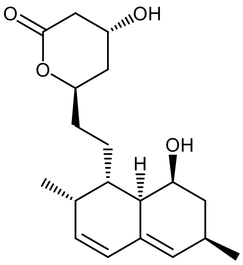

2. Monacolin J

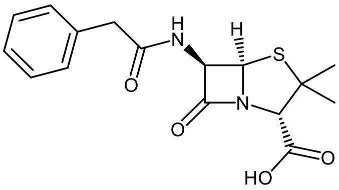

3. Penicillin G

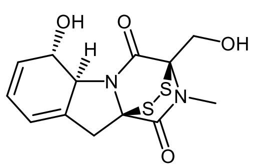

4. Gliotoxin

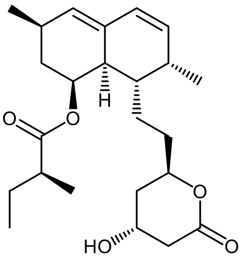

5. Lovastatin

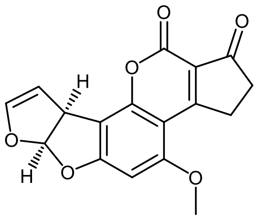

6. Aflatoxin B1

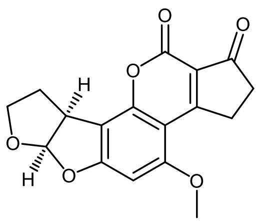

7. Aflatoxin B2

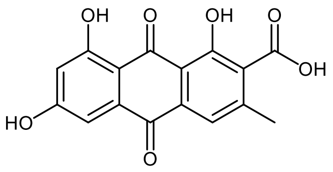

8. Endocrocin

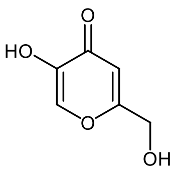

9. Kojic acid

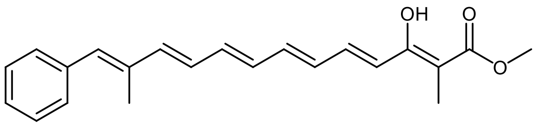

10. Asperrubrol

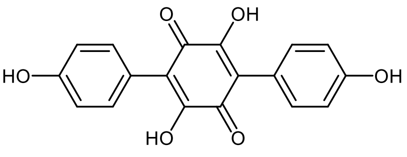

11. Atromentin

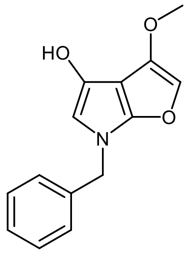

12. JBIR-86

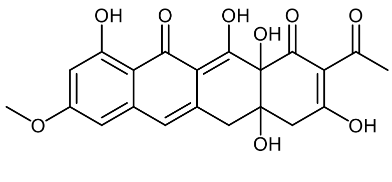

13. BMS-192548

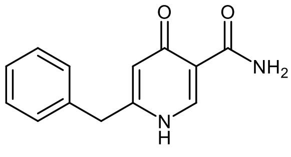

14. Aspernigrin A

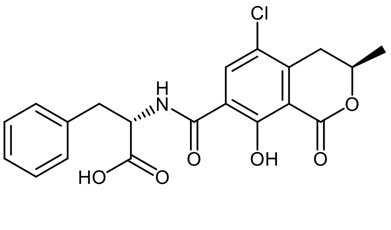

15. Ochratoxin A

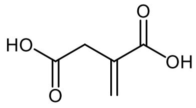

16. Itaconic acid

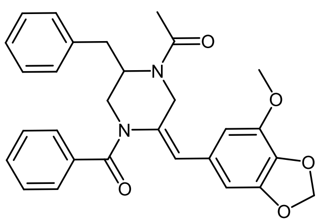

17. Flavipamide A

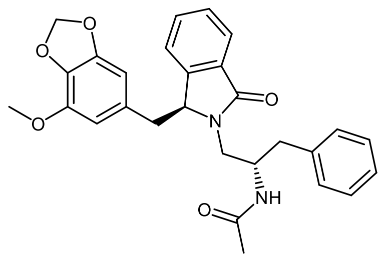

18. Flavipamide B

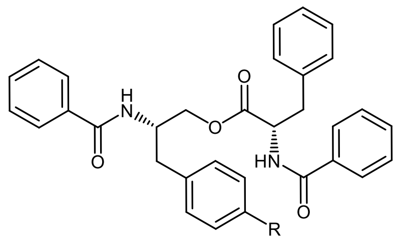

19. N-benzoylphenylalaniny-N-benzoylphenyl-alaninate R=H
20. 4′-OMe-asperphenamate R=OMe

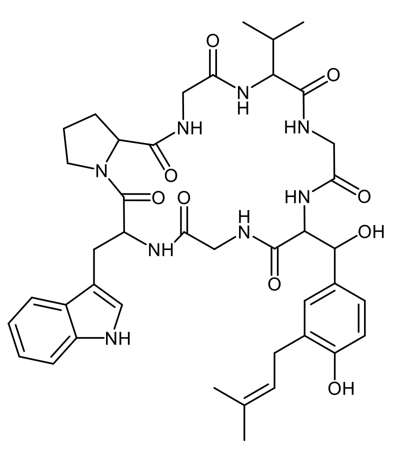

21. Cyclic Pro-Gly-Val-Gly-Try (/8-OH, 3-prenyl)-Gly-Trp

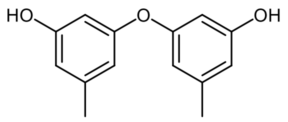

22. Diorcinol

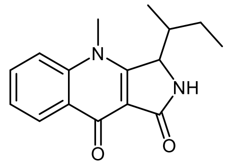

23. Quinolactacin A

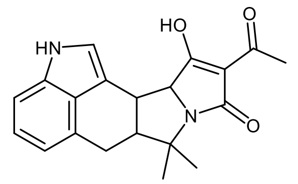

24. Cyclopiazonic acid

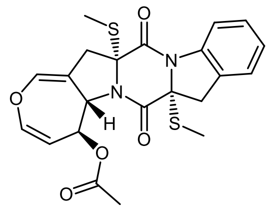

25. Versicolor A

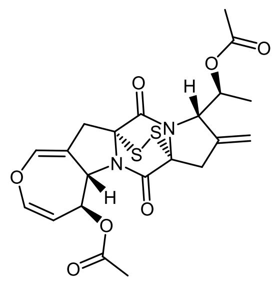

26. Acetylaranotin

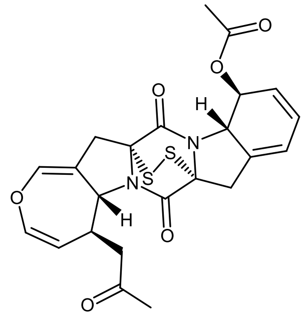

27. Acetylapoaranotin

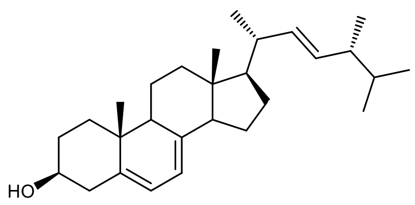

28. Ergosterol

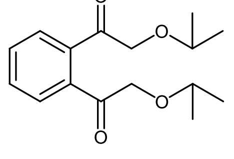

29. Diisobutyl phthalate

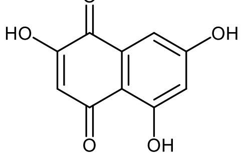

30. Flaviolin

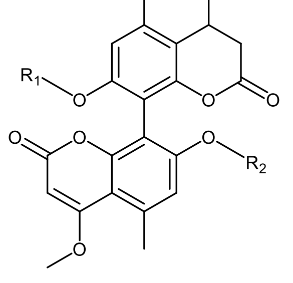

31. Orlandin R_1=H R_2=H
32 Kotanin R_1=CH_3 R_2=CH_3

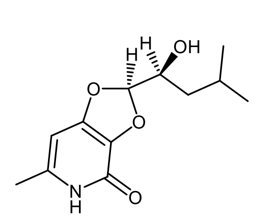

33. Dihydroisoflavipucines 1

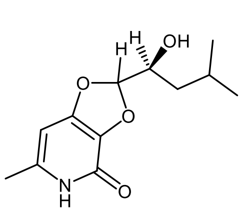

34. Dihydroisoflavipucines 2

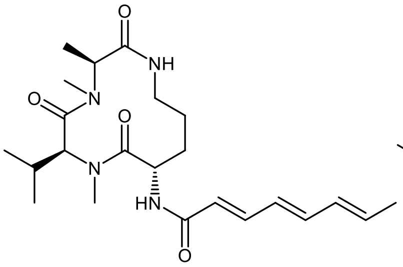

35. Aspochracin

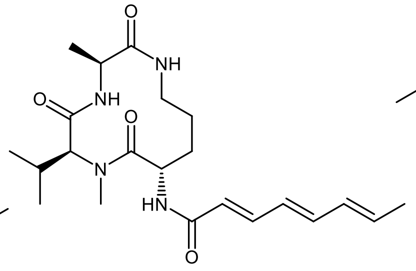

36. JBIR-15

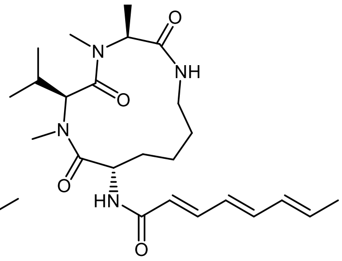

37. Sclerotiotide C

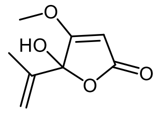

38. Penicillic acid

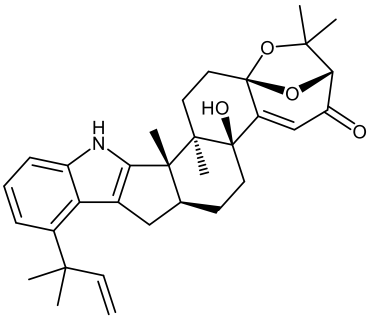

39. Aflatrem

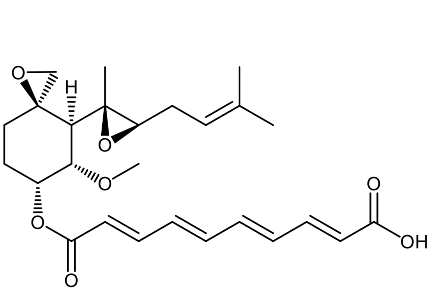

40. Fumagillin

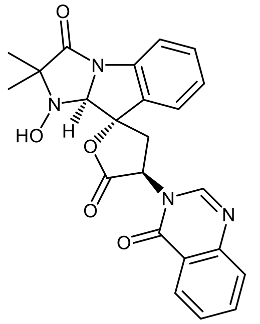

41. Fumitremorgin G

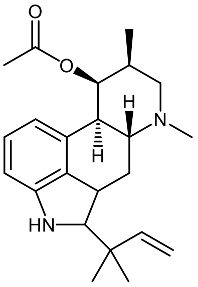

42. Fumigaclavine C

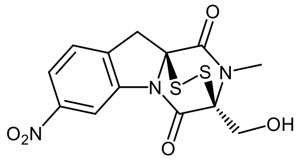

43. Glionitrin A

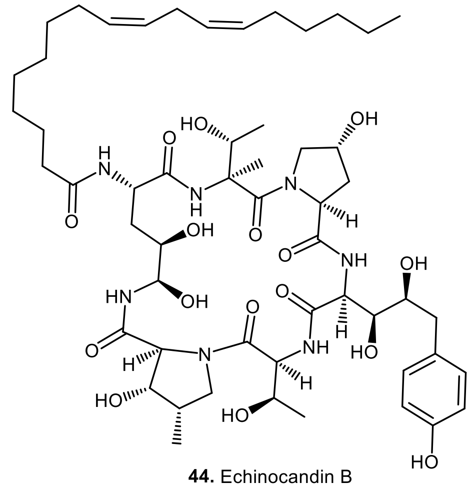

44. Echinocandin B

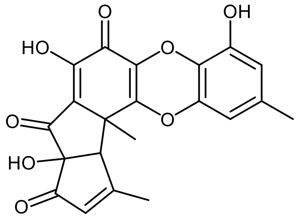

45. F9775A

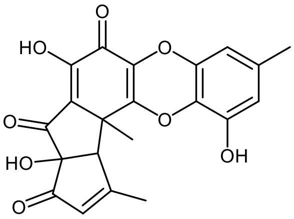

46. F9775B

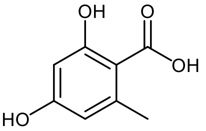

47. Orsellinic acid

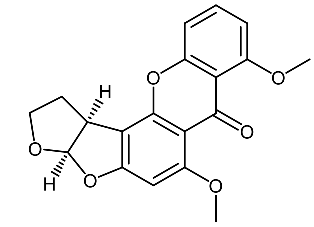

48. O-methylsterigmatocystin

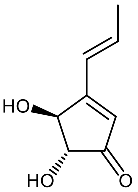

49. Terrein

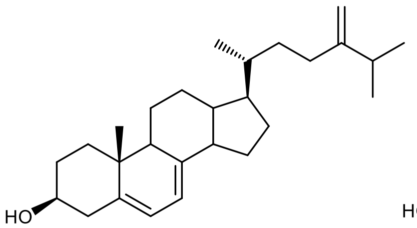

50. Ergosta-5,7,24(28)-trien-3β-ol

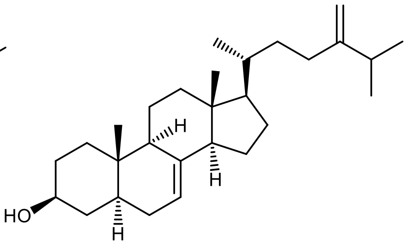

51. Episterol

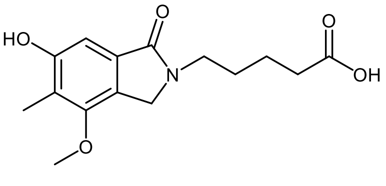

52. 1,3-Dihydro-6-hydroxy-4-meth-oxy-5-methy-1-oxo-2H-isoindole-2-pentanoic acid

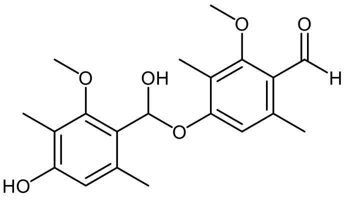

53. 4-[hydroxy(4-hydroxy-2-methoxy-3,6-dimethylphenyl)meth-oxy]-2-methoxy-3,6-dimethylbenzaldehyde

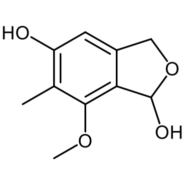

54. Cichorine intermediate

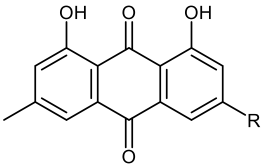

55. Emodin R=OH
56. Physcion R=OCH_3

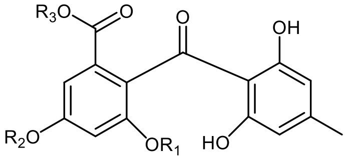

57. Sulochrin R_1=CH_3 R_2=H R_3=CH_3
58. 14-O-demethylsulochrin R_1=H R_2=CH_3 R_3=H

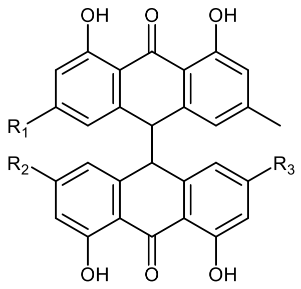

59. Physcion bianthrone R_1=OCH_3 R_2=CH_3 R_3=OCH_3
60. (trans)-emodin bianthrone R_1=OH R_2=CH_3 R_3=OH
61. (cis)-emodin bianthrone R_1=OH R_2=OH R_3=CH_3

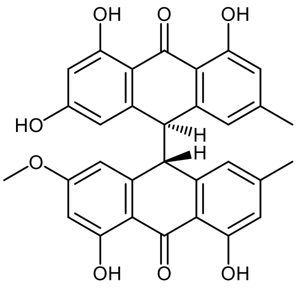

62. (trans)-emodin physcion bianthrone

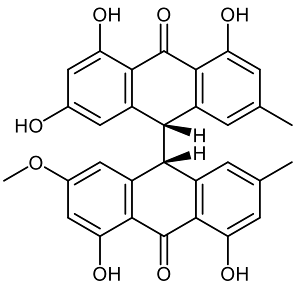

63. (cis)-emodin physcion bianthrone

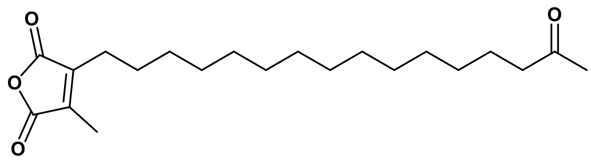

64. Aspergillus acid B

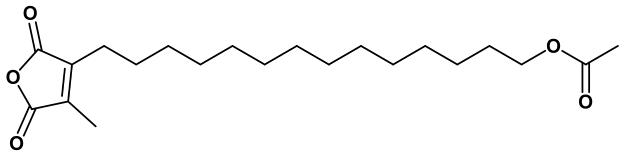

65. Aspergillus acid E

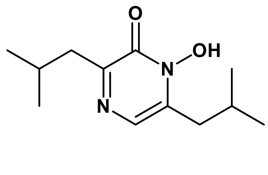

66. Neoaspergillic acid

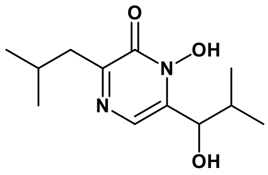

67. Neohydroxyaspergillic acid

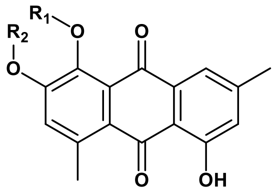

68. Gibellulin C R_1=CH_3 R_2=H
69. Gibellulin D R_1=H R_2=CH_3

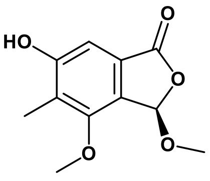

70. 3-methoxyporriolide

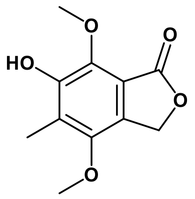

71. 7-methoxyporriolide

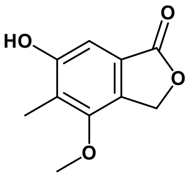

72. Porriolide

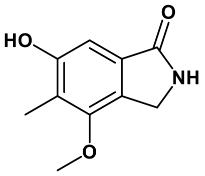

73. Cichorine

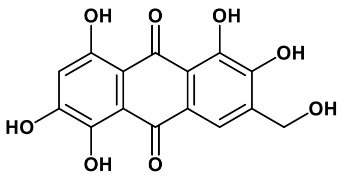

74. Asperthecin

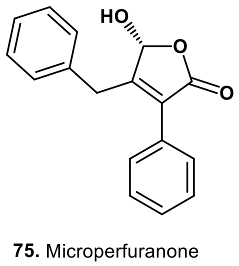

75. Microperfuranone

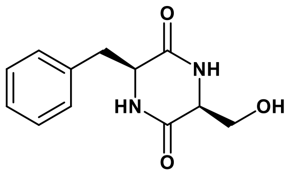

76. Cyclo(L-Phe-L-Ser)

Figure S1. Structures of secondary metabolite **1-76** regulated by global transcriptional regulators in *Aspergillus* spp.
